# Lipoprotein(a) and the risk of cardiovascular disease in the European population: results from the BiomarCaRE consortium

**DOI:** 10.1093/eurheartj/ehx166

**Published:** 2017-04-24

**Authors:** Christoph Waldeyer, Nataliya Makarova, Tanja Zeller, Renate B. Schnabel, Fabian J. Brunner, Torben Jørgensen, Allan Linneberg, Teemu Niiranen, Veikko Salomaa, Pekka Jousilahti, John Yarnell, Marco M. Ferrario, Giovanni Veronesi, Paolo Brambilla, Stefano G. Signorini, Licia Iacoviello, Simona Costanzo, Simona Giampaoli, Luigi Palmieri, Christa Meisinger, Barbara Thorand, Frank Kee, Wolfgang Koenig, Francisco Ojeda, Jukka Kontto, Ulf Landmesser, Kari Kuulasmaa, Stefan Blankenberg

**Affiliations:** 1 Department for General and Interventional Cardiology, University Heart Center Hamburg, Hamburg, Germany; 2 German Center for Cardiovascular Research (DZHK e.V.), partner site Hamburg/Lübeck/Kiel, Germany; 3 Faculty of Health and Medical Sciences, University of Copenhagen, Copenhagen, Denmark; 4 Research Centre for Prevention and Health, The Capital Region of Denmark, Glostrup, Denmark; 5 Medical Faculty, Aalborg University, Aalborg, Denmark; 6 Copenhagen University Hospital, Rigshospitalet, Copenhagen, Denmark; 7 National Institute for Health and Welfare, Helsinki, Finland; 8 Centre for Public Health, Queens University of Belfast, Belfast, Northern Ireland; 9 Department of Medicine and Surgery, Research Centre in Epidemiology and Preventive Medicine, University of Insubria, Varese, Italy; 10 Department of Medicina e Chirurgia, Università degli studi di Milano-Bicocca, Italy; 11 Department of Epidemiology and Prevention, Laboratory of Molecular and Nutritional Epidemiology, IRCCS Istituto Neurologico Mediterraneo Neuromed, Pozzilli, Isernia, Italy; 12 Istituto Superiore di Sanità, Rome, Italy; 13 Helmholtz Zentrum München, German Research Center for Environmental Health, Institute of Epidemiology II, Neuherberg, Germany; 14 Department of Internal Medicine II - Cardiology, University of Ulm Medical Center, Ulm, Germany; 15 Technical University of Munich, German Heart Center Munich, Munich, Germany; 16 German Center for Cardiovascular Research (DZHK e.V.), partner site Munich Heart Alliance, Munich, Germany; 17 Department of Cardiology, Charitè Universitötsmedizin Berlin, Campus Benjamin Franklin, Berlin, Germany; 18 Berlin Institute of Health (BIH), Berlin, Germany; 19 German Center for Cardiovascular Research (DZHK e.V.), partner site Berlin, Berlin, Germany

**Keywords:** Lipoprotein(a), Cardiovascular risk, Mortality, BiomarCaRE (Biomarker for Cardiovascular Risk Assessment in Europe), MORGAM (MONICA Risk Genetics Archiving and Monograph)

## Abstract

**Aims:**

As promising compounds to lower Lipoprotein(a) (Lp(a)) are emerging, the need for a precise characterization and comparability of the Lp(a)-associated cardiovascular risk is increasing. Therefore, we aimed to evaluate the distribution of Lp(a) concentrations across the European population, to characterize the association with cardiovascular outcomes and to provide high comparability of the Lp(a)-associated cardiovascular risk by use of centrally determined Lp(a) concentrations.

**Methods and results:**

Based on the Biomarkers for Cardiovascular Risk Assessment in Europe (BiomarCaRE)-project, we analysed data of 56 804 participants from 7 prospective population-based cohorts across Europe with a maximum follow-up of 24 years. All Lp(a) measurements were performed in the central BiomarCaRE laboratory (Biokit Quantia Lp(a)-Test; Abbott Diagnostics). The three endpoints considered were incident major coronary events (MCE), incident cardiovascular disease (CVD) events, and total mortality.

We found lower Lp(a) levels in Northern European cohorts (median 4.9 mg/dL) compared to central (median 7.9 mg/dL) and Southern European cohorts (10.9 mg/dL) (Jonckheere–Terpstra test *P* < 0.001). Kaplan–Meier curves showed the highest event rate of MCE and CVD events for Lp(a) levels ≥90^th^ percentile (log-rank test: *P *<* *0.001 for MCE and CVD). Cox regression models adjusted for age, sex, and cardiovascular risk factors revealed a significant association of Lp(a) levels with MCE and CVD with a hazard ratio (HR) of 1.30 for MCE [95% confidence interval (CI) 1.15‒1.46] and of 1.25 for CVD (95% CI 1.12‒1.39) for Lp(a) levels in the 67‒89th percentile and a HR of 1.49 for MCE (95% CI 1.29‒1.73) and of 1.44 for CVD (95% CI 1.25‒1.65) for Lp(a) levels ≥ 90th percentile vs. Lp(a) levels in the lowest third (*P *<* *0.001 for all). There was no significant association between Lp(a) levels and total mortality. Subgroup analysis for a continuous version of cube root transformed Lp(a) identified the highest Lp(a)-associated risk in individuals with diabetes [HR for MCE 1.31 (95% CI 1.15‒1.50)] and for CVD 1.22 (95% CI 1.08‒1.38) compared to those without diabetes [HR for MCE 1.15 (95% CI 1.08‒1.21; HR for CVD 1.13 (1.07–1.19)] while no difference of the Lp(a)- associated risk were seen for other cardiovascular high risk states. The addition of Lp(a) levels to a prognostic model for MCE and CVD revealed only a marginal but significant C-index discrimination measure increase (0.001 for MCE and CVD; *P *<* *0.05) and net reclassification improvement (0.010 for MCE and 0.011 for CVD).

**Conclusion:**

In this large dataset on harmonized Lp(a) determination, we observed regional differences within the European population. Elevated Lp(a) was robustly associated with an increased risk for MCE and CVD in particular among individuals with diabetes. These results may lead to better identification of target populations who might benefit from future Lp(a)-lowering therapies.

## Introduction

Lipoprotein(a) (Lp(a)) consists of apolipoprotein(a) and apolipoprotein B-100 and has a similar structure both to LDL-cholesterol and to plasminogen.^1^^,^^2^ Therefore, Lp(a) has a proatherogenic and a prothrombotic component, and is associated with the pathogenesis of cardiovascular (CV) disease.^3^^,^^4^ Elevated Lp(a) levels were proven as a marker of increased cardiovascular risk in numerous epidemiological and genetic studies during the past decades.^5–8^ Due to the lack of selective Lp(a)-lowering therapies the use of Lp(a) in clinical practice remains scarce. Recently, new compounds with the potential to lower Lp(a) like PCSK9-antibodies or the phase 2 study-proved IONIS-Apo(a) Rx, an antisense oligonucleotide targeting hepatic apo(a) mRNA,^9^^,^^10^ are presently evaluated in clinical trials. Therefore, the need for a precise characterization of the Lp(a)- associated CV risk is of increasing importance. For this purpose comparability of Lp(a) among different studies is essential.^11^^,^^12^ Although the largest meta-analysis revealed no significant differences among various methods of Lp(a) determination, it remains unclear whether the observed variability between studies was due to regional differences or due to the use of different assays.^6^ Further, underlying datasets should offer sufficient power to perform a diversity of subgroup analyses, in order to identify those individuals at highest Lp(a)-associated risk.

To achieve a more comparable as well as precise and timely characterization of the Lp(a)-associated CV risk in Europe we aimed to analyse the harmonized data of centrally measured Lp(a) of 7 cohorts from 5 European countries with 56 804 individuals and a maximum follow-up time of 24 years within the Biomarker for Cardiovascular Risk Assessment in Europe (BiomarCaRE) project.^13^

## Methods

### Study overview

The design and rationale of the BiomarCaRE project have been described previously in detail.^13^ Briefly, BiomarCaRE is based on the MORGAM (MONICA Risk Genetics Archiving and Monograph) Project.^14^ The MORGAM/BiomarCaRE Data Center in Helsinki harmonized individual data from 21 population-based cohort studies with central storage of selected biomaterial of more than 300 000 participants in the central BiomarCaRE laboratory in Hamburg. All presented Lp(a) concentrations were measured centrally at this laboratory site using the same Lp(a) assay. Local ethics review boards approved all participating studies.

### Study cohorts

The present analysis included data of 7 cohorts from 5 European countries comprising 56 804 individuals with available Lp(a) levels from 52 131 individuals. Cohorts involved were the FINRISK Study and the DanMONICA Study as Northern European cohorts, the Caerphilly Prospective Study and the Kooperative Gesundheitsforschung in der Region Augsburg (KORA) Study as Central European cohorts, and the MONICA Brianza Study, the MATISS cohort (Rome) and the Moli-Sani Project as Southern European cohorts. Each cohort is based on a well-defined population (see Supplementary material online, *Table S1*). Cohort descriptions are provided in Supplementary material online, *Box S1*.

For each cohort, the following harmonized variables were available at baseline: age, sex, body-mass-index (BMI), systolic blood pressure, lipid-lowering medication, anti-hypertensive medication, smoking status, history of diabetes, total cholesterol, low-density lipoprotein cholesterol (LDL), high-density lipoprotein cholesterol (HDL). LDL and HDL were not available for the Caerphilly Prospective Study. History of diabetes was defined as documented or self-reported, or diagnosed diabetes. Anti-hypertensive medication and smoking status were self reported. BMI, systolic blood pressure, and lipid parameters have been measured values. LDL cholesterol was calculated using the Friedewald formula.

### Study outcome

We defined the following outcome measures: (i) first fatal or non-fatal major coronary event (MCE) including the definite, possible, definite or possible (if not specifiable) acute myocardial infarction, coronary death, unstable angina pectoris, and cardiac revascularization, (ii) first major cardiovascular disease (CVD) event including the first fatal or non-fatal coronary heart disease event or likely cerebral infarction, coronary death, unstable angina pectoris, cardiac revascularization, ischaemic stroke, and unclassifiable death, and (iii) total mortality defined as mortality due to any cause during follow-up. Detailed endpoint definitions are in Supplementary material online.

### Laboratory procedures

All Lp(a) measurements were performed in the central BiomarCaRE laboratory between 2011 and 2015 using a fully automated, particle-enhanced turbidimetric immunoassay (Biokit Quantia Lp(a)-Test; Abbott Diagnostics, USA). This assay is not affected by apo(a) size heterogeneity.^15^ The limit of detection (LOD) is 0.38 mg/dL. Measurements are linear in the range of 1.3–90.0 mg/dL. Intra-assay coefficients of variation were <4% and inter-assay coefficients of variation were <9% for each cohort (see Supplementary material online, *Table S3*). The further included lipid parameters were measured locally at each participating centre.

### Statistical methods

Associations between baseline variables like total cholesterol, smoking status, hypertension, BMI, diabetes, sex (male), age at baseline examination, storage time, and Lp(a) concentrations were assessed using Spearman correlations. These were computed using mixed effects models combining individual participant data as described by Pigott and colleagues.^16^ This allowed us to consider cohort heterogeneity. Lp(a) concentrations >90 mg/dL were defined as 90 mg/dL.

For prediction of a first ever MCE, CVD event, and total mortality, only participants without a prior history of major CVD such as myocardial infarction (MI), hospitalized unstable angina, coronary artery bypass grafting, percutaneous transluminal coronary angioplasty, or ischaemic or haemorrhagic stroke were included.

Survival curves for MCE, CVD events, and total mortality were computed according to Lp(a) categories, using thirds and the 90th percentile, adapted from Kamstrup and colleagues.^11^ The last cut-off allowed to differentiate the risk for high and very high Lp(a) values. Furthermore, we performed additional analyses for Lp(a) levels ≥50 mg/dl vs.<50 mg/dL which is the suggested cut-off according to the ESC guidelines.^7^^,^^17^ These predefined categories were applied in the overall BiomarCaRE cohort.

Sex- and cohort-stratified Cox proportional hazards models for MCE, CVD events, and total mortality were computed using the individual level data from the available cohorts. For these analyses, Lp(a) was used after applying the cubic root transformation as a continuous variable and using pre-defined categories mentioned above. The Cox models for all three endpoints were adjusted in Model 1 for age as time scale, sex as strata and cohort as strata, and in Model 2 additionally for classical cardiovascular risk factors (smoking status, arterial hypertension, BMI, history of diabetes, total cholesterol, and age as time scale). We corrected LDL cholesterol and total cholesterol for the Lp(a) contribution according to the compositional data of Lp(a) with a cholesterol proportion of 30%.^18^ Therefore, Lp(a) was multiplied by 0.3 and this term was subtracted from LDL cholesterol and total cholesterol for each individual as done by previous studies.^11^ Since there was a substantial number of missing values regarding intake of lipid-lowering medication in the KORA and Caerphilly cohorts we did not adjust for this variable in the main outcome analysis but performed separate analyses with additional adjustment for intake of lipid-lowering medication in all cohorts except KORA and Caerphilly.

We classified subjects taking antihypertensive medication as being hypertensive. We examined the association between Lp(a) concentrations and time to event in different subgroups (age < 65 years vs. age ≥ 65 years, men vs. women, daily smoker vs. non-daily smoker, diabetes vs. no diabetes, hypertension vs. no hypertension, BMI < 30 vs. BMI ≥ 30, Southern, central Europe vs. Northern Europe, and LDL < 160 mg/dL vs. LDL ≥ 160 mg/dL). The previous models were extended by adding an interaction term between Lp(a) and subgroup indicator. This allowed to estimate subgroup specific Lp(a) hazard ratios and to test if the hazard ratios in the two categories of the subgroup variable were different. No adjustment for multiple testing was performed due to the exploratory nature of the analyses.^19^

The C-index^20^ and the net reclassification improvement (NRI)^21^^,^^22^ were used to quantify the added predictive value of Lp(a) beyond that from a model including classical risk factors. For the computation of NRI follow-up times were censored at ten years. Ten-fold cross validation was used to control for the over-optimism of calculating performance measures on the same dataset from which the models were computed. The risk categories used for the NRI analysis were <1%, 1 to <5%, 5 to <10%, and ≥10%^23^ for MCE, CVD, and total mortality. A version of NRI appropriate for survival analyses was computed using the Kaplan–Meier method.^22^ The overall NRI does not represent a proportion and is therefore reported as a decimal number between −2 and 2 rather than a percentage, as recommended by Leening and colleagues.^21^ Differences in C-statistics (with 95% CIs) after the addition of Lp(a) to the model consisting of cardiovascular risk factors were computed using the method described by Antolini and colleagues.^24^

A two-sided *P-*value of <0.05 was considered statistically significant. All statistical methods were implemented in R statistical software version 3.2.2 (www.R-project.org).

## Results

### Baseline characteristics

Baseline characteristics for the overall study population are shown in *Table **1*, of each individual cohort in Supplementary material online, *Table S1*, and according to predefined categories of Lp(a) levels in Supplementary material online, *Table S2.*

**Table 1:** Baseline characteristics of the study population

Characteristics	
Number of cohorts, *N*	7
Number of individuals, *N*	56 804
Years of baseline examinations, range in years	1986 − 2008
Men, *N* (%)	28 498 (50.2)
Women, *N* (%)	28 306 (49.8)
Age at baseline examination, *y*	52.4 (42.1, 62.0)
Cardiovascular risk factors	
Daily smokers, *N* (%)	13 304 (23.8)
Diabetes, *N* (%)	3063 (5.4)
Hypertension, *N* (%)	27 233 (48.1)
Body mass index, kg/m^2^	26.7 (24.0, 29.8)
Systolic blood pressure, mmHg	134.0 (121.0, 150.0)
Total cholesterol, mg/dL	215.0 (189.0, 245.0)
HDL cholesterol, mg/dL	54.0 (45.0, 65.0)
LDL cholesterol, mg/dL	122.0 (98.0, 147.0)
Medication	
Antihypertensive, *N* (%)	11 340 (20.3)
Cholesterol lowering, *N* (%)	2357 (5.2)
Lipoprotein (a)	
Information on lipoprotein (a), *N* (%)	52 131 (91.8)
Lipoprotein (a), mg/dL	8.7 (3.9, 19.1)
Endpoints during follow-up	
Major coronary event, *N* (%)	2452 (4.5)
Cardiovascular disease, *N* (%)	2966 (5.5)
Total mortality, *N* (%)	4877 (9.0)

Baseline characteristics are presented as absolute and relative frequencies for categorical variables, and quartiles for continuous variables as well as range in years for years of baseline examinations. Numbers of endpoints during follow-up are reported for individuals without CVD at baseline.

HDL, high density lipoprotein; LDL, low density lipoprotein.

Men and women were equally distributed (49.8%, *n* = 28 306 female subjects). The median age was 52.4 years. Study participants were slightly overweight (median BMI 26.7 kg/m^2^) and median systolic blood pressure was 134 mmHg. At baseline, 23.8% of the study cohort were daily smokers, 48.1% were diagnosed with hypertension, and 5.4% had diabetes. The median values for LDL-cholesterol, total cholesterol, and HDL-cholesterol at baseline were 122 mg/dL, 215 mg/dL, and 54 mg/dL, respectively.

### Distribution and correlations of Lp(a)

**Figure 1:**
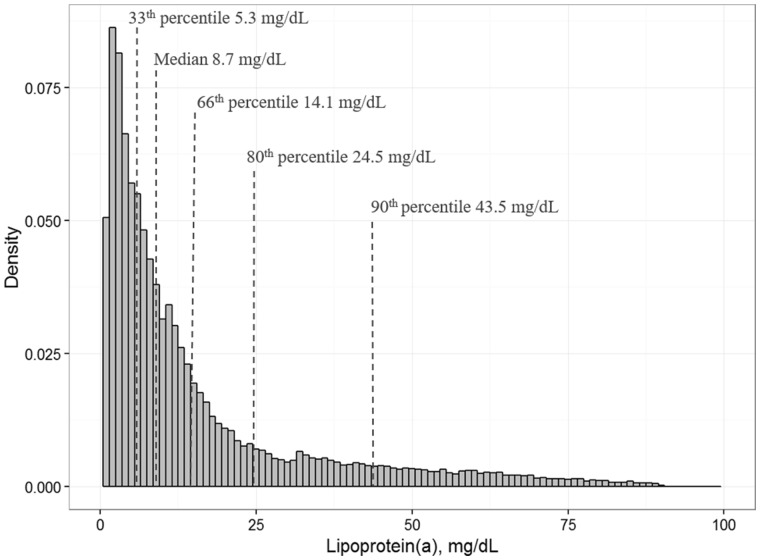
Density of Lp(a) levels in the entire study population. Density (y-axis) of Lp(a) levels (x-axis) in the entire study population including 52 131 measurements. Each column indicates the density of an Lp(a) range of 1 mg/dL. The median, 33th, 66th, 80th, and 90th percentiles are marked separately.

Lp(a) was measured in 91.8% (52 131 of 56 804) of study participants. The distribution of Lp(a) was skewed to the right in the overall cohort with a median of 8.7 mg/dL (IQR 3.9 to 19.1 mg/dL), a mean of 15.8 mg/dL [standard deviation (SD) 18 mg/dL] and the 66th, and 90th percentiles at 14.1 mg/dL and 43.5 mg/dL, respectively (*Figure **1*). We found a trend towards lower Lp(a) levels in the Northern European cohorts with an Lp(a) median of 4.9 mg/dL compared to an Lp(a) median of 10.9 mg/dL in Southern Europe (see Supplementary material online, *Figure S2*). To test if the gradient in median Lp(a) concentrations among North, Central, and South European populations is statistically significant, we performed Jonckheere–Terpstra test which resulted in *P*-value <0.001.^25^ Detailed illustrations of the distribution of Lp(a) of Northern, Central, and Southern European cohorts and for each particular cohort are shown in Supplementary material online, *Figure S1*.

In the overall cohort total cholesterol and age at baseline correlated positively with Lp(a) levels whereas male sex, diabetes, and BMI correlated negatively with Lp(a) levels. All correlations were only modest in nature (*Table **2*). Hypertension, smoking status, and storage duration as a technical variable did not correlate significantly with Lp(a) levels.

**Table 2:** Spearman correlations of Lp(a) and CV risk factors

	Total cholesterol	Daily smokers	Hyper- tension	BMI	Diabetes	Sex (male)	Age at baseline	Storage time
Correlation coefficient for Lp(a),	0.04	−0.02	0.01	−0.02	−0.02	−0.07	0.06	−0.01
*P*-value	*P* < 0.001	*P* = 0.061	*P* = 0.090	*P* = 0.0033	*P* < 0.001	*P* < 0.001	*P* < 0.001	*P* = 0.29

Spearman correlations of lipoprotein(a) with total cholesterol (Lp(a) corrected levels), smoker status, hypertension, body mass index, diabetes, age at baseline, and sex (male), and storage time. A linear mixed model was used to consider cohort heterogeneity.

BMI, body mass index.

### Outcome analysis

Two thousand four hundred and fifty-two incident MCE were observed during a median follow-up time of 8.8 years, 2966 incident CVD events after a median of 8.7 years, and 4877 deaths after a median of 9.2 years.

**Figure 2:**
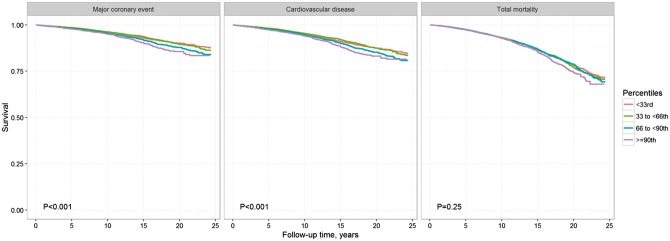
Kaplan–Meier curves according to predefined Lp(a) categories for the endpoints MCE, CVD events, and total mortality. The 33rd percentile of LP(a) corresponds to the value of 5.3 mg/dL, the 66th percentile corresponds to the value of 14.1 mg/dL, and the 90th percentile corresponds to the value of 43.5 mg/dL. *P*, *P*-value of log-rank test.

As illustrated in the Kaplan–Meier survival analyses across the predefined categories of Lp(a) levels MCE and CVD event rates increased with increasing Lp(a) levels with the highest event rates in the upper third of the distribution. No significant association between Lp(a) categories and all-cause mortality was observed (*Figure **2*).

**Figure 3:**
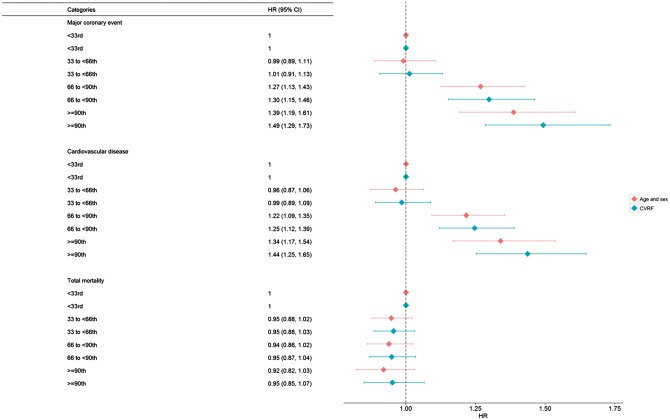
Cox regression analysis according to predefined Lp(a) categories (below 33rd percentile, 33rd-66th percentile, 67–89th percentile, above the 90th percentile) for the endpoints MCE, CVD events, and total mortality for two models of adjustment Model 1 (red rhombus) —adjusted for age, sex and cohort. Model 2 (blue rhombus)—adjusted for age, sex, cohort, smoking status, total cholesterol, diabetes, hypertension, and BMI. N events for MCE = 2038, N events for CVD events = 2478, and N events for total mortality = 3978. HR (95% CI), hazard ratio with 95% confidence interval.

Cox regression analyses revealed a significant association of Lp(a) with incident MCE such as Lp(a) levels between the 66th and 90th percentile were associated with a HR of 1.30 compared to the lowest third (95% CI 1.15–1.46) *P* < 0.01) after adjustment for a broad spectrum of risk factors (*Figure **3*, Models 1 and 2). Individuals with Lp(a) levels above the 90th percentile had the highest risk for future MCE with a HR of 1.49 (95% CI 1.29–1.73). Similar results were obtained when addressing a broader definition of CVD endpoints (*Figure **3*), whereas no significant association was found for total mortality. Further adjustment for lipid-lowering medication did not change the observed associations appreciably (data not shown).

Corresponding analyses for the clinically recommended threshold of Lp(a) ≥50 mg/dL vs. <50 mg/dL revealed comparable results for all investigated endpoints (see Supplementary material online, *Figures S3 and S4*). Cox regression analyses for Lp(a) treated as a continuous variable and the three outcome measures as well as regional stratified analysis by cohort and sorted by European region are displayed in Supplementary material online, *Figures S5 and S6*.

### Subgroup analysis of the Lp(a)-associated risk

**Figure 4:**
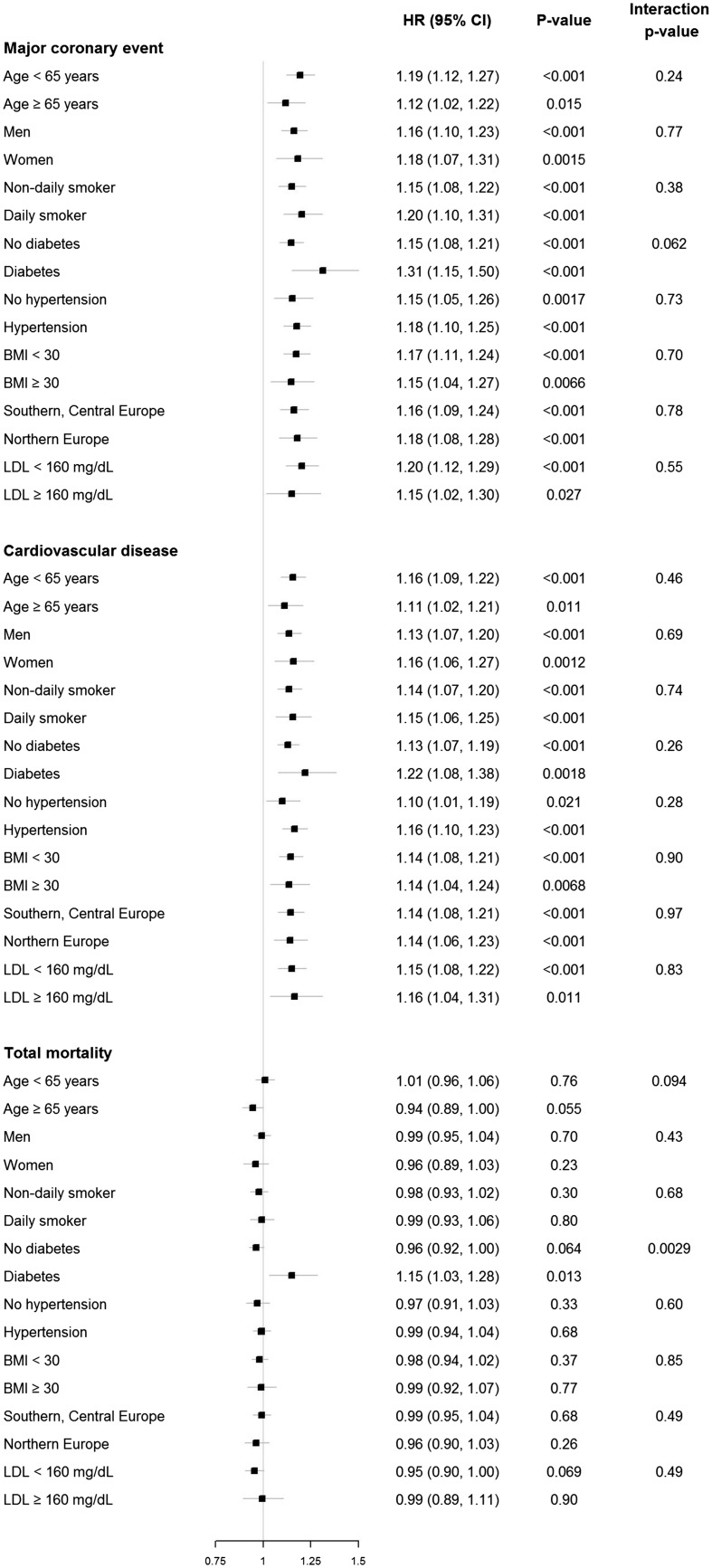
Subgroups analysis for a continuous version of cube root transformed Lp(a) for the endpoints MCE, CVD events, and total mortality. Subgroups used: age (<65 vs. ≥65 years), sex (men vs. women), smoking status, diabetes, hypertension, BMI (<30 vs. ≥30), European region (Southern, Central vs. Northern), and LDL (<160 mg/dL vs. ≥160mg/dL). HR (95%CI), hazard ratio (95% confidence interval), *P*, *P*-value for HRs.

Results of subgroup analyses of the Lp(a)-associated risk are shown in *Figure **4*. Hazard ratios for cube root transformed Lp(a) were comparable across various predefined subgroups except for individuals with diabetes. For these individuals, the Lp(a)-associated risk was higher (HR for MCE 1.31, for CVD 1.22, and for total mortality 1.15) compared to individuals without diabetes (HR for MCE 1.15, for CVD 1.13, and for total mortality 0.96). In individuals in other cardiovascular high risk states, e.g. smoking, hypertension, LDL ≥160 mg/dL or obesity (BMI ≥30 kg/m^2^) there was no relevant difference of the Lp(a)-associated risk compared to individuals without these cardiovascular high-risk states (*Figure **4*).

Importantly, despite regional differences of Lp(a) levels across Europe with lower Lp(a) levels in Northern European cohorts, the Lp(a)-associated risk in Northern European cohorts was comparable to the Lp(a)-associated risk in Central and Southern European cohorts.

### Lp(a) and prediction of major coronary events, cardiovascular disease events, and total mortality

Assessing C-statistics for prediction of MCE, CVD events, and total mortality we observed marginal but significant changes for MCE and CVD events and no changes for total mortality after the addition of Lp(a) to the base model (see Supplementary material online, *Table S4*).

Reclassification analyses after the addition of Lp(a) to a model consisting of cardiovascular risk factor (CVRF) variables are presented in Supplementary material online, *Table S5*. The addition of Lp(a) to the CVRF variables for MCE led to an NRI of 0.010 (95% CI −0.008 to 0.028), 0.006 (95% CI −0.011 to 0.023) for cases and 0.004 (95% CI 0.002 to 0.006) for non-cases. The addition of Lp(a) to the CVRFs algorithm for CVD events produced an NRI of 0.011 (95% CI from −0.006 to 0.028), 0.008 (95% CI −0.008 to 0.024) for cases and 0.003 (95% CI 0.001 to 0.005) for non-cases and almost no improvement for total mortality. Reclassification tables showing estimates of the expected number of reclassifications per risk category for cases and non-cases are provided in the Supplementary material online, *Table S5*.

## Discussion

Based on a harmonized large scale assessment of Lp(a) and cardiovascular outcome the main study findings are: (i) Lp(a) distribution has a north–south gradient with lower Lp(a) levels in Northern European populations compared to Central or Southern European populations. (ii) We confirm Lp(a) as a marker of cardiovascular risk in the European population with an particular increase of the Lp(a)-associated risk for MCE and CVD events above the 66th and the 90th percentile. (iii) The Lp(a)-associated risk was particularly observed in individuals with diabetes compared to those without diabetes.

### Regional distribution of Lp(a) levels across Europe

It is well known that Lp(a) levels vary strongly between ethnicities.^26^ However, because different Lp(a) assays lack precise comparability, differences of Lp(a) levels across individual participants from large-scale datasets have not yet been described in-depth. Our results showing lower Lp(a) levels in Northern European countries are consistent with a small cohort study measuring Lp(a) in 2164 participants older than 70 years from different regions of Europe.^27^ Furthermore, we observed a 0.582 fold lower Lp(a) median in the Finnish FINRISK cohort compared to the Lp(a) median in the German KORA cohort. Similar differences were seen between a German and another Finnish cohort by the group of Kronenberg *et al* (Florian Kronenberg, personal communication).

Lp(a) levels are mainly genetically determined by the number of kringle IV type 2 repeats which correlate strongly and inversely with Lp(a) levels whereas the influence of nutrition or lifestyle is rather weak.^28^ Further, Lp(a) levels are much higher in people with African ancestry compared to Caucasians. It is conceivable that over time inhabitants of Southern Europe have mixed more genetic characteristics with people of African origin than inhabitants of Northern Europe. Therefore, regional differences of Lp(a) levels in European populations might be due to lower numbers of kringle IV type 2 repeats in Southern European populations compared to Northern European populations. Interestingly, the regional differences of Lp(a) did not lead to differences regarding the Lp(a)-associated cardiovascular risk in different European regions. However, this issue has to be subject of further studies.

### Lp(a) as a marker of cardiovascular risk

Elevated Lp(a) levels have been demonstrated to be a marker of increased cardiovascular risk for a broad spectrum of subgroups. As the endpoint classification of CVD events was mainly driven by myocardial infarction, risk estimates for the endpoints CVD events and MCE are rather similar. The significant increase of the Lp(a)-associated risk for MCE and CVD events for Lp(a) levels above the 66th percentile is in line with previous studies. Also, the magnitude of the associations in our study is similar to others: The emerging risk factors collaboration meta-analysis found after adjustment for cardiovascular risk factors a HR of 1.27 for myocardial infarction or fatal coronary events for the top third vs. the lowest third of Lp(a) levels which is comparable to our HRs for MCE of 1.30 and 1.49 for Lp(a) levels in the 67–89th percentile and ≥90th percentile compared to the lowest third of Lp(a) levels.^6^ Using a lower-risk reference category of Lp(a)-levels <22nd percentile Kamstrup and colleagues found somewhat higher multivariable-adjusted HRs for myocardial infarction with 1.6 for Lp(a) levels in the 67–89th percentile, 1.9 for 90–95th percentile, and 2.6 for ≥95th percentile.^5^ In addition to the percentile-based analyses which are performed by the vast majority of Lp(a) studies we applied the clinically important threshold of 50 mg/dL and found a strong association for MCE and CVD events for Lp(a) levels ≥50 mg/dL compared to Lp(a) levels <50 mg/dL. This confirms the desirable Lp(a) level of <50 mg/dL recommended by the guidelines.^17^

The considerable size of the dataset allowed us to perform powerful subgroup analysis. The subgroup with the highest Lp(a)-associated cardiovascular risk were *n* = 2785 individuals with diabetes representing 5.4% of the total study population, while other cardiovascular high risk states did not influence the Lp(a)-associated cardiovascular risk. In previous studies, the results regarding the Lp(a)-associated risk in diabetic vs. non-diabetic individuals are heterogeneous or this issue has not be addressed.^6^^,^^29–31^ Since the presence of diabetes correlated negatively with Lp(a) levels in our analysis and in other studies, the increased risk in individuals with diabetes cannot be explained by higher Lp(a) levels in these individuals. Hence, there might be factors which induce a higher atherogenic or thrombogenic potency of Lp(a) e.g. due to glycosylation as known from LDL in a diabetic environment.^32^

In the present study, we found only slight NRI and C-Index change. The issue whether Lp(a) improves cardiovascular risk prediction has been addressed only by few studies yet. Comparability of reclassification analyses is often limited due to different base models and risk categories. Recently, Willeit and colleagues found a more relevant NRI and increase of the C-index for addition of Lp(a) to Framingham risk score and Reynolds risk score variables in 826 participants of the Bruneck study.^8^ Our data and results are rather comparable to the emerging risk factors collaboration analysis reporting only slight improvement of CVD prediction when adding Lp(a) to conventional CV risk factors in 165 544 participants from 37 cohorts.^33^ It is conceivable that in the emerging risk factors collaboration analysis and in our study residual confounding of large-scale data may have weakened the results compared to the more pronounced cardiovascular risk prediction in the small, very precisely characterized cohort of the Bruneck study. However, our results with only marginal risk prediction improvement confirm the guideline recommendation to determine Lp(a) not routinely in the setting of primary prevention.

### Comparison of Lp(a) levels to previous studies

Due to non-standardization of Lp(a)-assays the comparability of absolute Lp(a) levels in general is limited across different studies. Therefore, the largest meta-analysis of 26 cohort studies using a wide variety of different assays showed a range of Lp(a) medians between 3.0 and 23.0 mg/dL across the included studies.^6^ The present study, using the same assay in a central laboratory, revealed a range of cohort specific median values of Lp(a) between 4.6 and 11.3 mg/dL. One factor for the relatively low Lp(a) medians in our study could have been the prolonged storage duration of blood samples. However, the correlation of storage time as a technical variable and Lp(a) levels was only modest and statistically not significant. We noted comparable Lp(a) levels for cohorts within similar regions despite large differences in the storage duration (Brianza MONICA 25 years, Moli-Sani 8 years, MATISS 21 years) (see Supplementary material online, *Table S2*). Furthermore, the same Lp(a) assay used for the present study was evaluated in fresh plasma samples of a Northern Spanish population with a median of 25.3 nmol/L.^15^ Although the validity of a conversion from nmol/L to mg/dL is generally limited due to large differences of the molecular weight of Lp(a), applying the mean conversion factor of 2.4 suggested by Marcovina and colleagues^34^ to our measurements of stored samples results in a median of 26.1 nmol/L in the Southern European cohorts of the present study, which is comparable to the median of 25.3 nmol/L of Simo and colleagues in fresh samples of a Northern Spanish population.^15^

### Strengths and limitations

Some strengths and limitations of the present study merit consideration. Despite long-standing expertise in international standardization of data collection and data harmonization in the MORGAM Data Centre since 1984, resulting in excellent risk factor and endpoint validation, we cannot exclude a potential contribution of residual confounding for some of the observed effects among the more than 56 000 individuals investigated in 7 European population-based cohort studies. Of these, some variables are missing in some cohorts e.g. information on LDL and HDL values are not available for the Caerphilly cohort.

On the one hand we present a large scale dataset of Lp(a) measured centrally with the same assay, but on the other hand differences in storage duration among the included cohorts may have contributed to differences in the Lp(a) levels across populations. However, the resulting effect appears rather minor without a significant correlation for Lp(a) and storage duration. The leading cause for the remarkable differences across European population with lower Lp(a) levels in Northern European cohorts might be differences in the prevalence of kringle IV type 2 repeats. However, as we cannot provide genetic data for our analyses, this issue remains hypothetical and has to be addressed in future studies. Further, Lp(a) measurements were not performed consecutively so that we cannot correct for regression dilution bias. Despite the remarkable stability of Lp(a) this could have led to an underestimation of risk estimates.^35^

## Conclusion

In this large Lp(a) dataset on harmonized Lp(a) determination, we observed a north–south gradient of Lp(a) levels across Europe with lower Lp(a) levels in Northern European cohorts. Further, we could confirm Lp(a) as a marker of cardiovascular risk in the European population with an increasing cardiovascular risk for Lp(a)-levels above the 66th percentile. Individuals with diabetes had a particularly high Lp(a)-associated risk. These results may lead to better identification of target populations who might benefit most from future Lp(a)-lowering therapies.

## Supplementary material

Supplementary material is available at *European Heart Journal* online. 

## Funding

The BiomarCaRE Project is funded by the European Union Seventh Framework Programme (FP7/2007-2013) under grant agreement No. HEALTH-F2-2011-278913. The activities of the MORGAM Data Centre have been sustained by recent funding from European Union FP 7 project CHANCES (HEALTH-F3-2010-242244). A part of the biomarker determinations in the population cohorts was funded by the Medical Research Council London (G0601463, identification No. 80983: Biomarkers in the MORGAM Populations). The Open Access funding was provided by the BiomarCaRE Project.

## Ethics

Our study complies with the Declaration of Helsinki. The local ethics committees approved the research protocol. Informed consent has been obtained from the subjects (or their legally authorized representative).

**Conflict of interest:** Dr S.B. reports investigator-initiated grants from SIEMENS, Abbott Diagnostics and Thermofisher. Dr. W.K. reports receiving fees for serving on advisory boards from Novartis, Pfizer, The Medicines Company, Amgen, AstraZeneca, MSD, and Kowa, lecture fees from Amgen, AstraZeneca, Novartis, MSD, Sanofi, Actavis and berlin-Chemie as well as research grants from Roche, Beckmann, Singulex, and Abbott Diagnostics. Dr C.W. reports lecture fees from AstraZeneca. Dr U.L. received lecture or advisory fees from Amgen, Sanofi, MSD, Berlin-Chemie, Medicines Company, Abbott.

## Supplementary Material

Supplementary Tables and Figures
