# Supplementary material for: Lipoprotein(a) and the risk of cardiovascular disease in the European population: results from the BiomarCaRE consortium
Source: Eur Heart J. 2017 Apr 24;38(32):2490–8. doi: 10.1093/eurheartj/ehx166 (PMC5837491; doi:10.1093/eurheartj/ehx166)
Supplement: Supplementary Tables and Figures [file supplement_lpa_re_revised_28_02_2017_ehx166.pdf]

Table S1 Baseline characteristics of each participating cohort

**Characteristics**	**Brianza**	**Caerphilly**	**FINRISK**	**DanMONICA**	**KORA**	**Moli-Sani**	**MATISS**
Number of individuals, No.	4932	2171	8444	3530	8913	24325	4489
Years of baseline examinations, range in years	1986-1994	1990-1992	1997	1986-1991	1995-2000	2006-2008	1994-1995
Men, No. (%)	2432 (49.3)	2171 (100)	4253 (50.4)	1758 (49.8)	4427 (49.7)	11702 (48.1)	1755 (39.1)
Women, No. (%)	2500 (50.7)	0 (0)	4191 (49.6)	1772 (50.2)	4486 (50.3)	12623 (51.9)	2734 (60.9)
Age at baseline examination, years	46.7 (36.9, 56.0)	62.4 (58.5, 66.1)	48.7 (37.4, 59.6)	50.0 (39.8, 60.0)	50.5 (37.7, 61.9)	54.6 (45.8, 64.4)	50.3 (38.4, 61.7)
**Cardiovascular risk factors**							
Daily smoker, No. (%)	1479 (30.0)	542 (34.1)	1810 (21.9)	1496 (42.4)	2008 (22.5)	4949 (20.6)	1020 (22.7)
Diabetes, No. (%)	129 (2.7)	129 (5.9)	488 (5.8)	85 (2.4)	459 (5.1)	1576 (6.5)	197 (4.4)
Hypertension, No. (%)	1709 (34.9)	1295 (63.4)	3867 (45.8)	835 (23.7)	3566 (40.1)	13666 (56.2)	2295 (51.2)
Body-mass-index, kg/m²	24.9 (22.5, 27.8)	26.6 (24.4, 28.9)	26.2 (23.6, 29.2)	24.6 (22.3, 27.4)	26.5 (23.9, 29.6)	27.5 (24.7, 30.8)	27.5 (24.6, 30.6)
Systolic blood pressure, mmHg	128.0 (116.0, 141.0)	144.0 (130.0, 158.0)	134.0 (121.0, 149.0)	121.0 (111.0, 134.0)	130.0 (118.0, 144.0)	138.5 (125.5, 153.5)	136.0 (122.0, 153.0)
Total cholesterol, mg/dL	212.0 (183.0, 241.0)	239.8 (212.7, 266.8)	208.8 (185.6, 239.8)	224.3 (195.9, 255.2)	224.3 (197.2, 255.2)	211.0 (185.0, 239.0)	216.0 (189.0, 246.0)
HDL cholesterol, mg/dL	54.0 (46.0, 64.0)		52.2 (43.7, 62.6)	53.8 (44.5, 65.4)	53.4 (43.7, 65.7)	56.0 (47.0, 66.0)	50.0 (42.0, 59.0)
LDL cholesterol, mg/dL	115.0 (88.0, 146.0)		102.0 (82.0, 125.0)	144.0 (118.2, 172.7)	126.0 (104.0, 151.0)	125.0 (103.0, 148.0)	126.0 (101.0, 153.1)
**Medication**							
Antihypertensive, No. (%)	527 (10.7)	453 (22.3)	1134 (13.9)	240 (7.0)	1295 (14.5)	6894 (28.8)	797 (17.8)

Cholesterol lowering, No. (%)	42 (2.5)		279 (4.7)	11 (0.5)		1853 (8.0)	172 (3.8)
**Lipoprotein(a)**							
Information on lipoprotein (a), No. (%)	4549 (92.2)	1880 (86.6)	7790 (92.3)	3488 (98.8)	8048 (90.3)	22999 (94.5)	3377 (75.2)
Lipoprotein (a), mg/dL	10.0 (4.3, 20.7)	7.9 (3.4, 21.3)	4.6 (2.4, 10.3)	5.7 (2.6, 14.1)	7.9 (4.3, 18.5)	11.3 (5.2, 22.5)	9.4 (4.6, 19.9)
**Endpoints during follow-up**							
Major coronary event, No. (%)	281 (6.0)	492 (25.4)	513 (6.5)	405 (11.9)	332 (3.9)	331 (1.4)	98 (2.2)
Cardiovascular disease, No. (%)	355 (7.6)	492 (25.4)	705 (8.9)	489 (14.3)	423 (4.9)	371 (1.6)	131 (3.0)
Total mortality, No. (%)	535 (11.5)	1120 (57.9)	814 (10.3)	840 (24.6)	789 (9.2)	473 (2.0)	306 (7.0)

Baseline characteristics are presented as absolute and relative frequencies for categorical variables, and quartiles for continuous variables as well as ranges in years for years of baseline examinations. Lipoprotein (a) was measured using a fully automated, particle-enhanced turbidimetric immunoassay (Biokit Quantia Lp(a)-Test; Abbott Diagnostics, USA).

HDL stands for high density lipoprotein. LDL stands for low density lipoprotein. Numbers of endpoints during follow-up are reported for individuals without CVD at baseline.

Table S2 Baseline characteristics according to the predefined categories of Lp(a)

**Predefined categories of Lp(a), percentiles, (No.)** / **Characteristics**	**<33rd (N=15754)**	**33 to <66th (N=17821)**	**66 to <90th (N=13016)**	**≥90th (N=5540)**
Men, No. (%)	8639 (54.8)	8756 (49.1)	6015 (46.2)	2575 (46.5)
Women, No. (%)	7115 (45.2)	9065 (50.9)	7001 (53.8)	2965 (53.5)
Age at baseline examination, y	50.5 (40.5, 60.6)	51.7 (41.8, 61.6)	54.1 (43.9, 63.2)	53.6 (43.6, 63.2)
**Cardiovascular risk factors**				

Daily smoker, No. (%)	3883 (25.0)	4302 (24.5)	2848 (22.2)	1191 (21.9)
Diabetes, No. (%)	930 (5.9)	930 (5.3)	659 (5.1)	266 (4.8)
Hypertension, No. (%)	7350 (46.7)	8248 (46.4)	6537 (50.3)	2789 (50.5)
Body-mass-index, kg/m²	26.6 (23.9, 29.9)	26.6 (23.9, 29.7)	26.9 (24.1, 30.0)	26.7 (24.1, 29.8)
Systolic blood pressure, mmHg	133.5 (120.5, 149.0)	133.5 (121.0, 148.5)	135.5 (122.5, 150.5)	135.5 (122.0, 151.0)
Total cholesterol, mg/dL	208.8 (181.7, 239.8)	212.7 (186.0, 242.0)	220.0 (193.0, 248.0)	227.0 (201.0, 255.2)
HDL cholesterol, mg/dL	52.2 (43.0, 63.0)	54.0 (45.0, 64.6)	55.7 (47.0, 66.0)	56.0 (48.0, 66.0)
LDL cholesterol, mg/dL	125.3 (102.4, 149.4)	128.8 (106.8, 153.6)	135.5 (112.7, 160.5)	140.0 (117.6, 164.8)
**Medication**				
Antihypertensive, No. (%)	2874 (18.5)	3409 (19.4)	2961 (23.1)	1241 (22.7)
Cholesterol lowering, No. (%)	491 (4.6)	633 (5.0)	677 (6.9)	327 (8.1)
**Lipoprotein (a)**				
Information on Lp(a), No.	15754	17821	13016	5540
Lp(a), mg/dL	2.6 (1.8, 3.6)	8.1 (6.3, 10.4)	20.8 (16.1, 29.5)	57.9 (49.1, 68.1)
**Endpoints during follow-up**				
Major coronary event, No. (%)	901 (5.7)	819 (4.6)	649 (5.0)	314 (5.7)
Cardiovascular disease, No. (%)	1107 (7.0)	958 (5.4)	759 (5.8)	360 (6.5)
Total mortality, No. (%)	1768 (11.2)	1664 (9.3)	1068 (8.2)	497 (9.0)

Baseline characteristics are presented as absolute and relative frequencies for categorical variables, and quartiles for continuous variables as well as ranges in years for years of baseline examinations. HDL stands for high density lipoprotein. LDL stands for low density lipoprotein. Numbers of endpoints during follow-up are reported for individuals without CVD at baseline.

Table S3 Coefficients of variation and mean storage duration for each cohort

	**Coefficients of variation (%)**		**Mean storage duration, years**
**Cohort**	intra-assay	inter-assay	
**DanMONICA**	3.2	5.3	20.0
**FINRISK**	1.3	6.6	15.7
**Caerphilly**	2.7	6.8	19.4
**KORA**	1.1	6.9	19.8
**Brianza**	1.1	8.3	25.3
**Moli Sani**	2.1	6.5	7.1
**MATISS**	2.0	7.7	21.5

Table S4 C-Indices for all investigated endpoints

Pattern A

C-index (CVRFs) (base model)	C-index (CVRFs) + LP(a)	C-index difference to base model; p-value
0.84 (0.82, 0.85)	0.84 (0.82, 0.85)	0.001 (0.000, 0.002); 0.043

Pattern B

C-index (CVRFs) (base model)	C-index (CVRFs) + LP(a)	C-index difference to base model; p-value
0.83 (0.81, 0.84)	0.83 (0.82, 0.85)	0.001 (0.000, 0.002); 0.034

Pattern C

C-index (CVRFs) (base model)	C-index (CVRFs) + LP(a)	C-index difference to base model; p-value
0.82 (0.81, 0.84)	0.82 (0.81, 0.84)	0.000 (0.000, 0.000); 0.77

C-Indices for **(A)** major coronary events, **(B)** cardiovascular disease events, and **(C)** total mortlity. For all endpoints the cardiovascular risk factor (CVRF) variables were used to adjust the models. If no Lp(a) was used in the model these variable define the 'base model'. Age was used as the time scale of the Cox models (so they are implicitly adjusted for age). A Weibull baseline hazard was used to compute the event probabilities (from the Cox models). These (10 years) event probabilities were used to compute the C-indices.

Table S5 Net reclassification improvement for all investigated endpoints

Pattern A

CASES	<1%	1 to <5%	5 to <10%	≥10%	Reclassified up, No. (%)	Reclassified down, No. (%)	NRI
**CVRFs and lipoprotein (a)**					(95% CI)		
<1%	47	5	0	0	93 (4.8)	81 (4.2)	0.006 (-0.011, 0.023)
1 to <5%	7	436	30	0			
5 to <10%	0	20	495	58			
≥10%	0	0	53	843			
**NONCASES**							
<1%	17 644	386	0	0	1 206 (2.6)	1 398 (3.0)	0.004 (0.002, 0.006)
1 to <5%	508	15 985	463	0			
5 to <10%	0	533	5 544	357			
≥10%	0	0	358	4 253			
**OVERALL**							**0.010 (-0.008, 0.028)**

Pattern B

CASES	<1%	1 to <5%	5 to <10%	≥10%	Reclassified up, No. (%)	Reclassified down, No. (%)	NRI
**CVRFs and lipoprotein (a)**					(95% CI)		
<1%	31	9	0	0	107 (4.8)	91 (3.9)	0.008 (-0.008, 0.024)
1 to <5%	5	395	35	0			
5 to <10%	0	31	534	63			
≥10%	0	0	55	1 171			
**NONCASES**							
<1%	14 638	363	0	0	1 137 (2.5)	1 263 (2.8)	0.003 (0.001, 0.005)
1 to <5%	387	16 294	417	0			
5 to <10%	0	493	6 247	384			
≥10%	0	0	383	5 474			
**OVERALL**							**0.011 (-0.006, 0.028)**

Pattern C

CASES	<1%	1 to <5%	5 to <10%	≥10%	Reclassified up, No. (%)	Reclassified down, No. (%)	NRI
**CVRFs and lipoprotein (a)**					(95% CI)		
<1%	118	1	0	0	5 (0.2)	8 (0.3)	-0.001 (-0.004, 0.002)
1 to <5%	1	372	1	0			
5 to <10%	0	1	443	3			

≥10%	0	0	6	2 155			
**NONCASES**							
<1%	15 603	41	0	0	138 (0.3)	167 (0.4)	0.000 (0.000, 0.001)
1 to <5%	62	14 707	42	0			
5 to <10%	0	54	6 297	55			
≥10%	0	0	51	8 019			
**OVERALL**							**0.000 (-0.004, 0.003)**

Net reclassification improvement by endpoint with estimates of the expected number of reclassifications per risk category for cases and noncases. **(A)** for major coronary events, **(B)** for cardiovascular disease events, and **(C)** for total mortality. NRI is presented as a number with a theoretical range between -2 and 2.

Figure S1 Geographical overview of the included BiomarCaRE cohorts and the according European regions with the corresponding Lp(a) medians in mg/dL

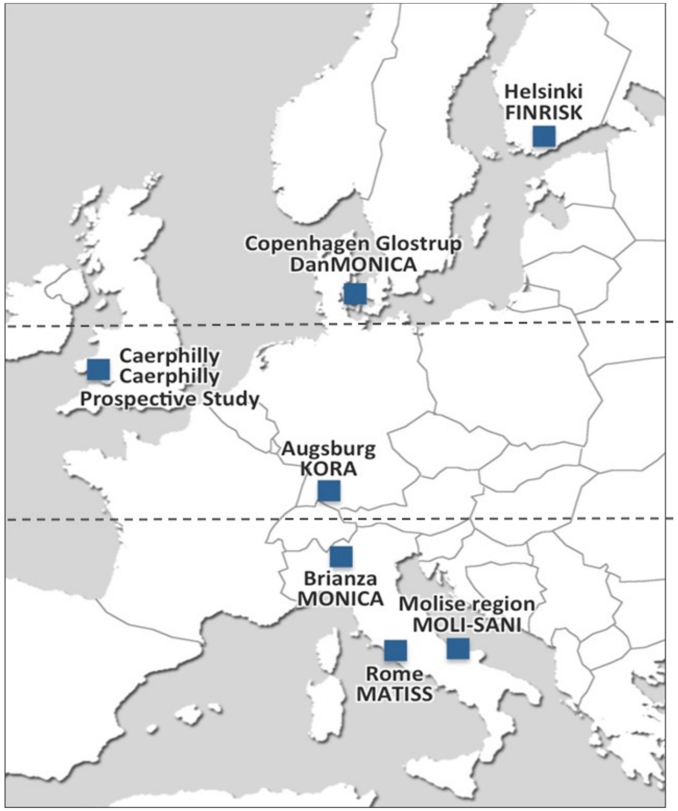

	Median Lp(a) (IQR) mg/dL
**FINRISK**, Finland	4.6 (2.4; 10.3)
**DanMONICA**, Denmark	5.7 (1.5; 7.8)
= **Northern Europe**	**4.9**
**Caerphilly**, Wales	7.9 (3.4; 21.3)
**KORA**, Germany	7.9 (4.3; 18.5)
= **Central Europe**	**7.9**
**Brianza MONICA**, Italy	10.0 (4.3; 20.7)
**Moli-Sani**, Italy	11.3 (5.2; 22.5)
**MATISS,** Italy	9.4 (4.6, 19.9)
= **Southern Europe**	**10.9**

Jonckheere-Test, *P*-value <0.001. IQR stands for interquartile range.

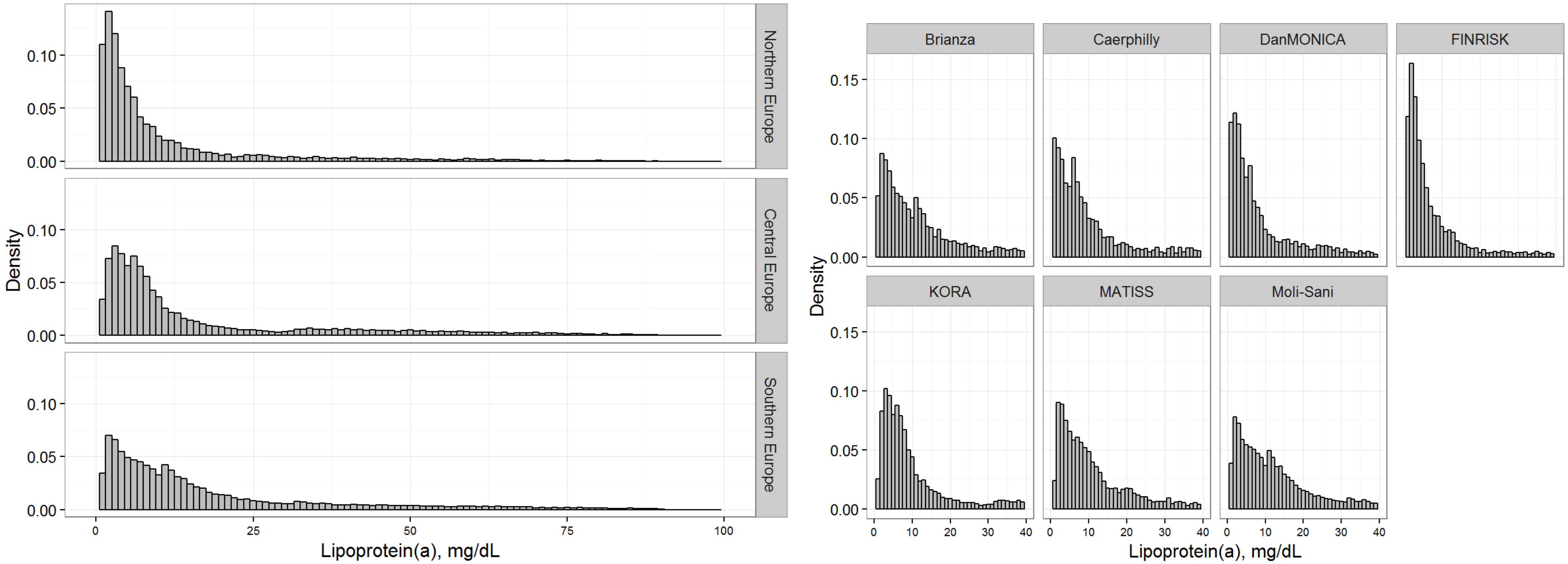

Figure S2 Density of Lp(a) levels for European regions and for each particular cohort

Density (y-axis) of Lp(a) levels (x-axis) **(A)** in the Northern European, Central European, and Southern European cohorts and **(B)** in each particular cohort.

Figure S3: Kaplan-Meier curves according to the Lp(a) categoriy <50 mg/dL and ≥50 mg/dL for the endpoints major coronary events, CVD events, and total mortality

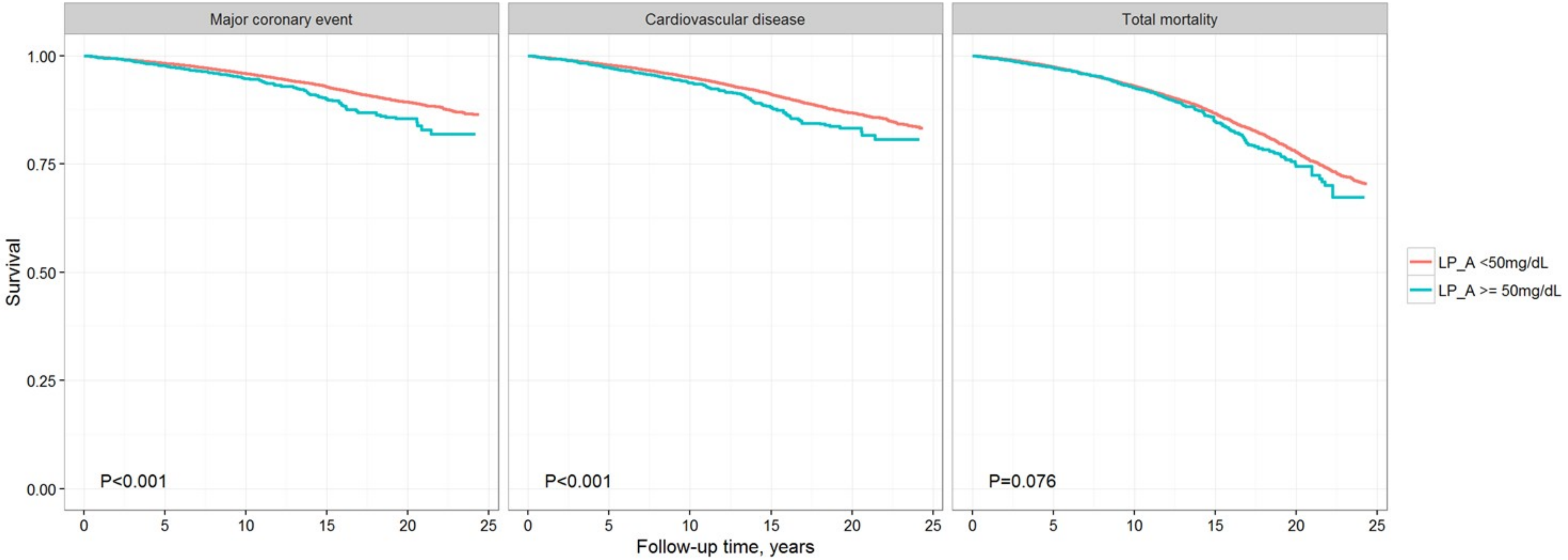

P stands for *P*-value of log-rank test.

Figure S4: Cox regression analysis according to the Lp(a) categoriy <50 mg/dL and ≥50 mg/dL for the endpoints major coronary events, CVD events, and total mortality

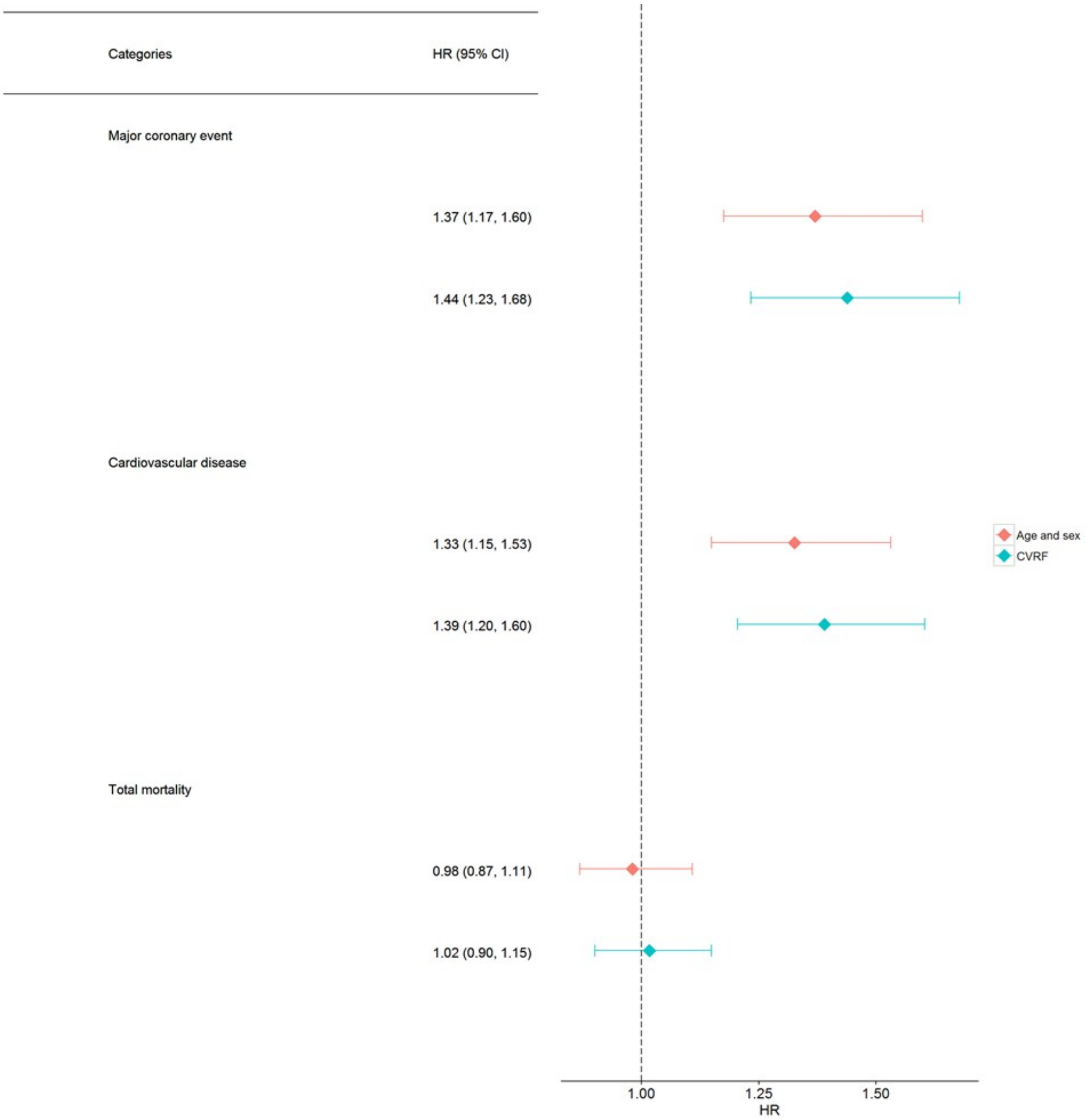

Adjusted for age and sex (model1 ♦) and for age, sex, smoking status, total cholesterol, diabetes, hypertension and BMI (model 2 ♦). N events for MCE = 2038, N events for CVD events = 2478 N events for total mortality = 3978. HR (95%CI) stands for hazard ratio (95% CI stands for confidence interval).

Figure S5 Cox regression analysis for a continuous version of cube root transformed Lp(a) for all investigated endpoints

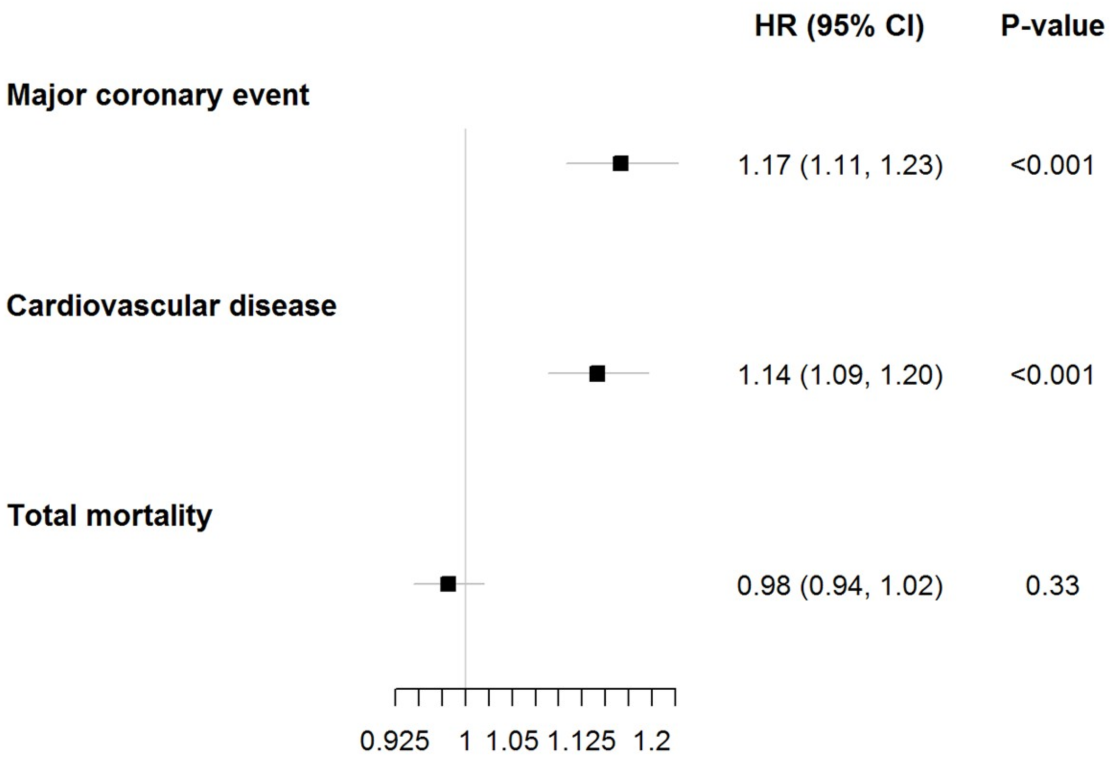

Cox regression analysis for a continuous version of cube root transformed Lp(a) for the endpoints major coronary event, CVD event, and total mortality adjusted for age, sex, smoking status, total cholesterol, diabetes, hypertension and BMI. HR (95%CI) stands for hazard ratio (95% confidence interval).

Figure S6 Cox regression analysis for a continuous version of cube root transformed Lp(a) for the endpoints major coronary events, CVD events, and total mortality

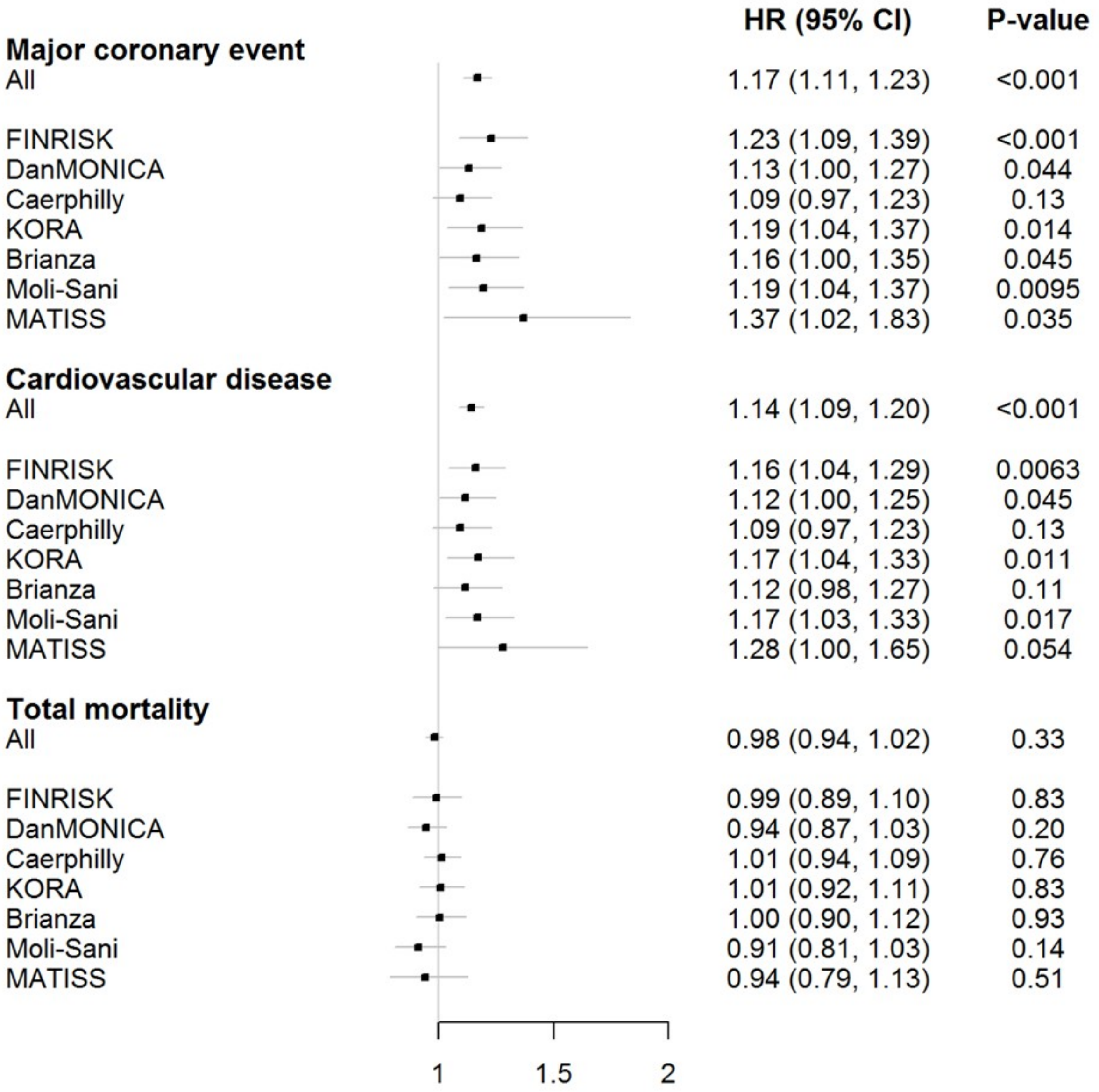

Stratified by cohort and sorted by European region (see Supplementary figure S1). Adjusted for age, sex, smoking status, total cholesterol, HDL cholesterol, diabetes, hypertension and BMI. HR (95%CI) stands for hazard ratio (95% confidence interval).
